# Cancer incidence in sites potentially related to occupational exposures: 58 years of follow-up of firefighters in the Norwegian Fire Departments Cohort

**DOI:** 10.5271/sjweh.4009

**Published:** 2022-03-31

**Authors:** Niki Marjerrison, Jarle Jakobsen, Tom K Grimsrud, Johnni Hansen, Jan Ivar Martinsen, Karl-Christian Nordby, Marit B Veierød, Kristina Kjærheim

**Affiliations:** 1 Department of Research, Cancer Registry of Norway, Oslo, Norway; 2 Oslo Centre for Biostatistics and Epidemiology, Department of Biostatistics, Institute of Basic Medical Sciences, University of Oslo, Oslo, Norway; 3 Danish Cancer Society Research Center, Copenhagen, Denmark; 4 National Institute of Occupational Health, Oslo, Norway

**Keywords:** carcinogen, cohort study, firefighting, occupational epidemiology

## Abstract

**Objectives:**

Firefighters are exposed to a variety of known and suspected carcinogens through their work. However, the association with cancer risk has limited evidence. We examined cancer incidence among firefighters in the newly established Norwegian Fire Departments Cohort restricted to sites with established associations with carcinogens encountered during firefighting. This included sites within the respiratory, urinary, and lympho-hematopoietic systems, and the skin and all sites combined.

**Methods:**

Male firefighters (N=3881) in the cohort were linked to the Cancer Registry of Norway for incident cancer cases occurring during the period 1960–2018. We calculated standardized incidence ratios (SIR) with rates for the national male population as reference, and stratified SIR analyses by period of first employment, duration of employment, and time since first employment.

**Results:**

Elevated risk was seen for all sites combined (SIR 1.15, 95% confidence interval 1.07–1.23). Elevated risk of urinary tract cancer was observed among firefighters who began working before 1950, and with observation ≥40 years since first employment. Risk of mesothelioma and laryngeal cancer were elevated with ≥40 years since first employment and with ≥30 years employment duration.

**Conclusions:**

The observed associations between firefighting and urinary tract cancer, laryngeal cancer, and mesothelioma have been observed in some studies previously, and our results suggest the observed elevated risks are related to carcinogenic occupational exposures. Differences in risk by period of employment potentially reflect changes in exposures from improved quality and use of personal protective equipment.

Firefighters can be exposed to a variety of known and suspected carcinogens through their work. At the fire scene, substances classified as Group 1 human carcinogens by the International Agency for Research on Cancer (IARC) may be present, such as benzene, polycyclic aromatic hydrocarbons (PAH), polychlorinated biphenyls (PCB), 1,3-butadiene, 2,3,7,8-tetrachlorodibenzo-*para*-dioxin, asbestos, and formaldehyde (1–3). Firefighters’ equipment can contain hazardous chemicals such as plasticizers (4) which when exposed to heat, may lead to the release of carcinogenic substances. Equipment can also become contaminated with carcinogens at the fire scene (5, 6), subsequently also contaminating the fire station (7). Both at the fire scene and station, firefighters can be exposed to carcinogenic diesel exhaust (1, 8). In addition to exposure to known carcinogens, simultaneous exposure to a multiplicity of chemicals not recognized as carcinogens alone have also been hypothesized to contribute to carcinogenic effects (9).

Inhalation and dermal absorption of carcinogens are the predominant exposure routes relevant to firefighters (1). Post-fire, elevated levels of PAH have been detected on the neck (3, 5), hands (5, 10), and in the urine (11–13) of firefighters. Post-fire breath samples have also detected elevated levels of benzene and other volatile organic compounds (3, 11, 14). Positive-pressure self-contained breathing apparatus (SCBA) offer firefighters a workplace protection factor of about 10 000 (15). However, systemic exposures have been detected even when SCBA was used, suggesting that exposure to carcinogens via dermal absorption and off-gassing from contaminated equipment can still occur (3, 11, 14). Further, in Norway, SCBA is not yet widely used through all stages of firefighting (16).

Exposure to carcinogens relevant to firefighters have been associated with sufficient evidence in humans with increased risk of various cancer sites within the respiratory tract, skin, urinary organs and lympho-hematopoietic system, as well as to all sites combined (17, 18). Among these sites, recent meta-analyses of firefighter studies have reported elevated incidence of bladder cancer (19, 20), cutaneous melanoma (20), and mesothelioma (19). Again, among sites with established associations with relevant carcinogens, a study on Nordic firefighters found elevated incidence of lung adenocarcinoma, mesothelioma, cutaneous melanoma, non-melanoma skin cancer, and all sites combined (21).

In 2007, an IARC working group classified occupational exposure as a firefighter as possibly carcinogenic (1). Based on the epidemiological evidence, the working group identified testicular cancer, prostate cancer, and non-Hodgkin lymphoma as the sites most consistently associated with firefighting. Carcinogenic exposures relevant to firefighting were reviewed, but existing epidemiological studies did not in general include information on such specific exposures. Thus, the precise role between firefighters’ specific occupational exposures and cancer risk remains unclear.

The aim of our study was to assess cancer incidence in sites of *a-priori* interest based on established associations with carcinogenic exposures normally encountered during firefighting by linking firefighters in the newly established Norwegian Fire Departments Cohort to outcome registries.

## Methods

### The Norwegian Fire Departments Cohort

The Norwegian Fire Departments Cohort was established between 2017 and 2019 in cooperation with firefighting departments and firefighters’ unions. With the intent of including all geographic regions and as many of the largest professional fire departments in Norway as possible, 21 fire departments were invited to participate in the cohort. Of those invited, 14 accepted, and 1 additional department self-selected. As of 2019, these 15 participating fire departments provided firefighting services for nearly 50% of the Norwegian population (16), and the geographical distribution of the participating fire departments reflected that of the general population.

All individuals who worked at the participating departments between 1950 and 2019 were sought to be included in the cohort. Data based on personnel records were recorded by fire department staff, partly assisted by research assistants. For each person, the following information was entered into a database designed for that purpose: birth date, national personal identification number, vocational education, department and station(s) at which they worked, positions and time periods for each position held, and whether or not the position involved smoke-diving.

**Figure 1:**
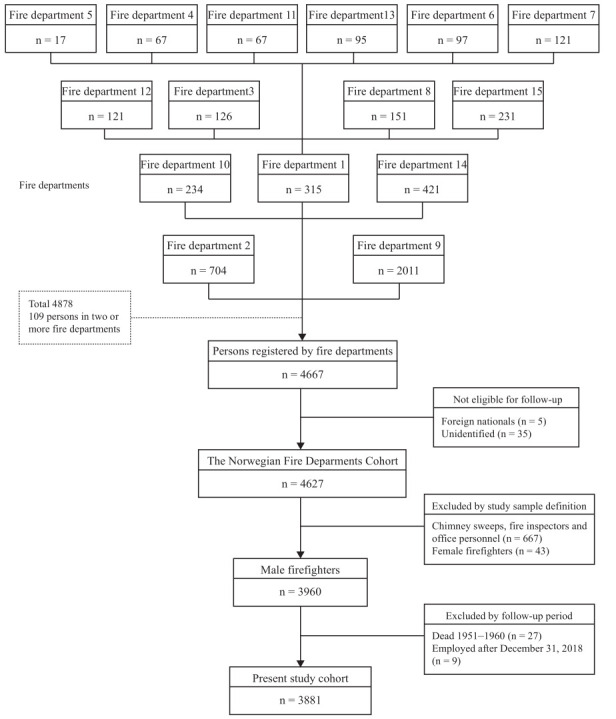
Flowchart of the study cohort of male firefighters in the Norwegian Fire Departments Cohort, 1950–2018.

The participating departments registered 4667 persons (figure 1). Those who could not be identified by their personal identification number (N=35) and foreign nationals (N=5) were excluded because they could not be followed up in national registries. Ultimately, the Norwegian Fire Departments Cohort included 4627 persons.

### Study sample

Of the 4627 persons in the cohort, we excluded non-firefighting personnel with positions only as chimney sweeps, fire inspectors, or office personnel (N=667). Female firefighters (N=43) were excluded from the present analyses due to their low numbers. Those who died before 1960 (N=27) or were first employed after 2018 (N=9) were excluded based on the study follow-up period (1 January 1960–31 December 2018). Thus, the final study sample included 3881 men with positions entailing present or former active firefighting.

Employment duration for each individual was calculated as the time from the beginning of their first employment period until the end of their final employment period or end of follow-up. For those missing the date but not year of their earliest registered employment period (N=3), 1 July of the registered year was used. For those missing the end date of their latest registered employment period, the earliest of the following was used: the first of the month in which the individual reached age 65 years (N=49), date of death (N=11), date of emigration (N=2), or end of follow-up (N=1226).

### Follow-up

A person entered follow-up on the latter of 1 January 1960 or start of first employment, and was followed until the first date of emigration, death, or 31 December 2018. The cohort was linked to national outcome registries using the personal identification number given to all Norwegian citizens alive in 1960 or born later; therefore, follow-up began in 1960. Date of emigration was obtained from the Norwegian Population Register. Cause and date of death were obtained from the Cause of Death Registry. Date and diagnosis of cancer cases were obtained from the Cancer Registry of Norway (CRN). There has been mandatory reporting of all cancer cases in Norway since the start of the registry in 1952, and the degree of completeness and accuracy is considered high (22). Cancer diagnoses from the CRN are updated and classified according to the 10^th^ revision of the International Classification of Diseases (ICD-10) for the codes C00–C96.

The present study assessed the risk of cancer for sites with an established association with carcinogenic exposures that firefighters can face. Exposures were identified from the IARC Monograph (volume 98) on firefighting (1), and cancer sites associated with these exposures with sufficient evidence in humans were identified from a summary of carcinogenic associations (18). This included respiratory tract cancers (lung, larynx, and mesothelioma), cutaneous melanoma, non-melanoma skin cancer (excluding basal cell carcinoma), urinary tract cancer, and cancers in the lympho-hematopoietic system. We assessed kidney cancer alongside urinary tract cancers because of their close physiological association within the urinary system. We assessed the aforementioned sites – having established associations with carcinogenic exposures – combined in a group named “exposure-associated sites”, as well as all sites combined. Cancers for which an increased risk is often seen among firefighters but evidence of association with known occupational exposures in humans is limited, such as prostate cancer, were not included in the analyses (18).

### Statistical analysis

Standardized incidence ratios (SIR) were calculated as the ratio of the observed and expected number of cases, with the rates of the general Norwegian male population as the reference. Person-years in five-year age and one-year calendar period strata were multiplied with the respective reference rates to obtain the number of expected site-specific or overall cancer cases. The exact 95% confidence intervals (CI) were calculated assuming a Poisson distribution of the observed number of cases.

Three time-based stratifications were conducted to examine nuanced trends in cancer risk: by year of first employment (static; <1950, 1950–1969 and ≥1970), to reflect how occupational carcinogenic exposures have changed over time; by employment duration (dynamic; <10, 10–19, 20–29, and ≥30 years), as a proxy for cumulative exposure; and by time since first employment (dynamic; <20, 20–39 and ≥40 years) to account for the latency period of cancer.

All analyses were conducted using Stata 16 (Stata Corp, College Station, TX, USA).

## Results

The 3881 men who had worked in positions entailing active firefighting at any of the 15 participating fire departments in Norway accrued a total of 108 358 person-years, with an average of 27.9 years of follow-up (table 1). Among them, there were 845 incident cancer cases. Year of birth ranged from 1885 to 1996, and start of employment from 1913 to 2018 (supplementary material, www.sjweh.fi/article/4009, figure S1). The mean age at first employment was 27.6 years, and the average duration of employment was 21.8 years, with 74% having ≥10 years of registered employment (supplementary figure S2). Of the firefighters, 103 (3%) had worked at ≥1 department, and 92% worked full time throughout their registered employment; 51 (1%) had employment periods only for positions entailing ≤5% of full time employment.

**Table 1:** Characteristics of the study sample, male firefighters in the Norwegian Fire Departments Cohort (N=3881). Follow-up from 1 January 1960 to 31 December 2018.

Characteristics	N (SD)	%
Person-years at risk	108 358	
Mean years of follow-up	27.9 (16.1)	
Status on December 31, 2018		
Emigrated	27	0.7
Dead	1105	28.5
Alive	2749	70.8
Year of birth		
<1950	1542	39.7
1950–1969	1147	29.6
≥1970	1192	30.7
Age at first employment (years)		
<30	2928	75.4
30–49	920	23.7
≥50	33	0.9
Year of first employment		
<1950	655	16.9
1950–1969	574	14.8
1970–1989	1144	29.5
≥1990	1508	38.8
Duration of employment (years)		
<10	1006	25.9
10–19	683	17.6
20–29	716	18.5
30–39	1362	35.1
≥40	114	2.9

In the overall analysis (table 2), the highest risk was observed for mesothelioma (SIR 2.47, 95% CI 0.99–5.06). Cutaneous melanoma and cancers of the larynx, kidney, and urinary tract tended to occur more frequently than expected. For the exposure-associated sites taken together, SIR was 1.10 (95% CI 0.98–1.22), and for all sites combined the SIR was 1.15 (95% CI 1.07–1.23).

**Table 2:** Observed number of cases and standardized incidence ratios (SIR) with 95% confidence intervals (CI) for selected cancer sites and all cancers combined among male firefighters (N=3881) in the Norwegian Fire Departments Cohort. Follow-up from 1 January 1960 to 31 December 2018. [ICD=International Classification of Diseases].

Cancer site	ICD-10	Observed	SIR	95% CI
Larynx	C32	12	1.77	0.91–3.08
Lung	C33-C34	81	0.98	0.78–1.22
Cutaneous melanoma	C43	47	1.30	0.95–1.73
Non-melanoma skin ^a^	C44	35	0.99	0.69–1.37
Mesothelioma	C45	7	2.46	0.99–5.06
Kidney ^b^	C64	29	1.28	0.86–1.84
Urinary tract ^c^	C65-C68	69	1.25	0.97–1.58
Hodgkin lymphoma	C81	2	0.53	0.06–1.91
Non-Hodgkin lymphoma	C82-C86, C96	26	1.17	0.76–1.71
Multiple myeloma	C90	9	0.79	0.36–1.51
Leukaemia	C91-C95	14	0.83	0.46–1.40
Exposure-associated sites ^d^		331	1.10	0.98–1.22
All cancers	C00-C96	845	1.15	1.07–1.23

a Excluding basal cell carcinoma.

b Excluding renal pelvis.

c Including bladder and renal pelvis.

d Includes the following sites: C32, C33-C34, C43, C44, C45, C64, C65-C68, C81, C82-C86, C90, C91-C95, C96

In analyses stratified by year of first employment, we observed elevated incidence of urinary tract cancer among those who began working before 1950 (SIR 1.71, 95% CI 1.19–2.38) (table 3). With later period of first employment, the SIR for urinary tract cancer decreased, and a similar trend was observed for kidney and lung cancer. For exposure-associated sites combined, the most prominently elevated risk was observed among those who began working before 1950 (SIR 1.36, 95% CI 1.14–1.62). For all sites combined, SIR was also highest among those who began working before 1950 (1.29, 95% CI 1.15–1.44).

**Table 3:** Stratification of observed number of cases and standardized incidence ratios (SIR) with 95% confidence intervals (CI) for selected cancer sites and all cancers combined among male firefighters (N=3881) in the Norwegian Fire Departments Cohort. Follow-up from 1 January 1960 to 31 December 2018. [ICD= International Classification of Diseases; Obs=observed; pyr=person-years].

Cancer site	Year of first employment							

	<1950 (N=655)		1950–1969 (N=574)		≥1970 (N=2652)			
		
	Obs	SIR (95% CI)	Obs	SIR (95% CI)	Obs	SIR (95% CI)		
Larynx	6	2.34 (0.86–5.09)	5	2.02 (0.65–4.71)	1	0.57 (0.01–3.18)		
Lung	40	1.37 (0.98–1.87)	28	0.87 (0.58–1.26)	13	0.61 (0.33–1.04)		
Cutaneous melanoma	8	1.38 (0.59–2.71)	19	1.53 (0.92–2.38)	20	1.11 (0.68–1.72)		
Non-melanoma skin ^a^	9	0.72 (0.33–1.37)	17	1.10 (0.64–1.76)	9	1.20 (0.55–2.28)		
Mesothelioma	3	3.74 (0.77–10.9)	2	1.52 (0.18–5.49)	2	2.74 (0.33–9.90)		
Kidney ^b^	10	1.61 (0.77–2.96)	9	1.24 (0.57–2.35)	10	1.09 (0.52–2.01)		
Urinary tract ^c^	35	1.71 (1.19–2.38)	22	1.04 (0.65–1.58)	12	0.88 (0.45–1.54)		
Hodgkin lymphoma	0	0.00 (0.00–3.75)	2	2.29 (0.28–8.28)	0	0.00 (0.00–1.42)		
Non-Hodgkin lymphoma	6	1.14 (0.42–2.47)	9	1.20 (0.55–2.27)	11	1.17 (0.58–2.09)		
Multiple myeloma	5	1.21 (0.39–2.82)	1	0.25 (0.01–1.40)	3	0.93 (0.19–2.71)		
Leukaemia	5	0.91 (0.29–2.11)	4	0.72 (0.20–1.84)	5	0.88 (0.29–2.05)		
Exposure-associated sites ^d^	127	1.36 (1.14–1.62)	118	1.07 (0.89–1.28)	86	0.93 (0.74–1.15)		
All cancers	304	1.29 (1.15–1.44)	284	1.08 (0.96–1.22)	257	1.08 (0.95–1.22)		
	
Cancer site	Time since first employment							

	<20 years (52 093 pyr)		20–39 years (41 034 pyr)		≥40 years (15 230 pyr)			
		
	Obs	SIR (95% CI)	Obs	SIR (95% CI)	Obs	SIR (95% CI)		

Larynx	0	0.00 (0.00–7.04)	2	0.59 (0.07–2.14)	10	3.33 (1.60–6.13)		
Lung	4	1.07 (0.29–2.74)	22	0.64 (0.40–0.98)	55	1.23 (0.93–1.60)		
Cutaneous melanoma	9	1.33 (0.61–2.53)	21	1.36 (0.84–2.08)	17	1.21 (0.70–1.94)		
Non-melanoma skin ^a^	3	2.14 (0.44–6.26)	8	0.97 (0.42–1.96)	24	0.93 (0.59–1.38)		
Mesothelioma	0	0.00 (0.00–30.4)	1	0.98 (0.02–5.46)	6	3.47 (1.27–7.55)		
Kidney ^b^	1	0.47 (0.01–2.64)	15	1.41 (0.79–2.32)	13	1.32 (0.70–2.26)		
Urinary tract ^c^	3	1.13 (0.23–3.30)	17	0.86 (0.50–1.38)	49	1.49 (1.10–1.97)		
Hodgkin lymphoma	0	0.00 (0.00–1.70)	0	0.00 (0.00–2.19)	2	3.05 (0.37–11.0)		
Non-Hodgkin lymphoma	4	1.30 (0.35–3.32)	14	1.50 (0.82–2.52)	8	0.81 (0.35–1.61)		
Multiple myeloma	0	0.00 (0.00–4.31)	4	0.88 (0.24–2.26)	5	0.82 (0.27–1.91)		
Leukaemia	1	0.48 (0.01–2.70)	6	0.92 (0.34–1.99)	7	0.86 (0.34–1.77)		
Exposure-associated sites ^d^	25	1.01 (0.65–1.49)	110	0.96 (0.79–1.16)	196	1.25 (1.08–1.44)		
All cancers	66	1.09 (0.84–1.39)	314	1.12 (1.00–1.25)	465	1.18 (1.08–1.29)		
	
Cancer site	Duration of employment							

	<10 years (33 405 pyr)		10–19 years (26 539 pyr)		20–29 years (24 930 pyr)		≥30 years (23 483 pyr)	
			
	Obs	SIR (95% CI)	Obs	SIR (95% CI)	Obs	SIR (95% CI)	Obs	SIR (95% CI)

Larynx	0	0.00 (0.00–5.55)	2	2.70 (0.33–9.75)	1	0.51 (0.01–2.85)	9	2.53 (1.16–4.80)
Lung	4	0.62 (0.17–1.59)	7	0.86 (0.34–1.76)	18	0.81 (0.48–1.29)	52	1.14 (0.85–1.49)
Cutaneous melanoma	10	1.84 (0.88–3.38)	5	0.85 (0.27–1.98)	13	1.38 (0.73–2.35)	19	1.23 (0.74–1.92)
Non-melanoma skin ^a^	3	1.02 (0.21–2.98)	5	1.56 (0.51–3.63)	7	0.83 (0.34–1.72)	20	0.96 (0.58–1.48)
Mesothelioma	1	4.21 (0.11–23.4)	0	0.00 (0.00–11.4)	1	1.38 (0.03–7.66)	5	3.09 (1.00–7.20)
Kidney ^b^	3	1.32 (0.27–3.85)	3	1.07 (0.22–3.14)	6	0.95 (0.35–2.06)	17	1.51 (0.88–2.42)
Urinary tract ^c^	8	1.82 (0.79–3.60)	3	0.55 (0.11–1.60)	22	1.54 (0.97–2.34)	36	1.16 (0.81–1.60)
Hodgkin lymphoma	0	0.00 (0.00–2.46)	0	0.00 (0.00–3.63)	0	0.00 (0.00–3.64)	2	2.17 (0.26–7.85)
Non-Hodgkin lymphoma	2	0.72 (0.09–2.61)	4	1.28 (0.35–3.27)	10	1.68 (0.81–3.10)	10	0.96 (0.46–1.77)
Multiple myeloma	1	1.07 (0.03–5.97)	0	0.00 (0.00–2.47)	4	1.32 (0.36–3.39)	4	0.65 (0.18–1.66)
Leukaemia	2	1.02 (0.12–3.70)	2	0.94 (0.11–3.38	0	0.00 (0.00–0.69)	10	1.20 (0.57–2.20)
Exposure-associated sites ^d^	34	1.17 (0.81–1.63)	31	0.91 (0.62–1.30)	82	1.06 (0.84–1.32)	184	1.18 (1.02–1.37)
All cancers	74	1.01 (0.79–1.27)	87	1.06 (0.85–1.31)	217	1.15 (1.00–1.32)	467	1.19 (1.09–1.30)

a Excluding basal cell carcinoma.

b Excluding renal pelvis.

c Including bladder and renal pelvis.

d Includes the following sites: C32, C33-C34, C43, C44, C45, C64, C65-C68, C81, C82-C86, C90, C91-C95, C96

In analyses stratified by time since first employment, with observation to ≥40 years, we observed elevated incidence of laryngeal cancer (SIR 3.33, 95% CI 1.60–6.13), mesothelioma (SIR 3.47, 95% CI 1.27–7.55), and urinary tract cancer (SIR 1.49, 95% CI 1.10–1.97) (table 3). Lung cancer, in the stratum 20–39 years since first employment, was the only site with significantly fewer observed cases than expected (SIR 0.64, 95% CI 0.40–0.98), but SIR >1 were observed in the other two strata. For exposure-associated sites combined, we observed elevated risk in the stratum with ≥40 years since first employment (SIR 1.25, 95% CI 1.08–1.44). For all sites combined, SIR increased up to 1.18 (95% CI 1.08–1.29) in the stratum with ≥40 years since first employment.

In analyses stratified by duration of employment, we observed elevated incidence of laryngeal cancer with an employment duration ≥30 years (SIR 2.53, 95% CI 1.16–4.80) (table 3). Incidence of mesothelioma was also elevated with employment duration ≥30 years (SIR 3.09, 95% CI 1.00–7.20). For urinary tract cancers, there were more cases than expected for all lengths of employment except for 10–19 years (only three cases). For exposure-associated sites combined, we observed elevated incidence with ≥30 years employment duration (SIR 1.18, 95% CI 1.02–1.37). For all sites combined, we observed an expected incidence for the shortest employment duration group, and increasing SIR with increasing duration up to 1.19 (95% CI 1.09–1.30) with employment duration ≥30 years.

## Discussion

This study evaluated cancer incidence in sites of *a-priori* interest based on established associations with known carcinogenic exposures which can occur during firefighting. The cohort included 3881 male firefighters, and through a follow-up of 58 years, overall cancer incidence was modestly elevated for all sites combined compared with the general male population. Elevated risk of urinary tract cancer was found among those who started as a firefighter before 1950 and those with ≥40 years since first employment. Increased incidence of mesothelioma and laryngeal cancer was observed among those with ≥30 years employment duration and ≥40 years since first employment.

### Lung and laryngeal cancer

Lung and laryngeal cancers were of *a-priori* interest based on the risk of inhalation of toxic smoke during firefighting activities. Overall, we observed near-expected incidence of lung cancer and non-significantly elevated incidence of laryngeal cancer. Surprisingly, meta-analyses have also found near-expected incidence of these cancers previously (19, 20, 23). Stratifications revealed that the numbers of observed cases of laryngeal and lung cancer were higher than expected with an employment duration ≥30 years and with ≥40 years since first employment, though risk estimates were highest among those with first employment before 1950 and decreased thereafter, potentially pointing towards period-dependent differences in exposure.

Occupational exposures to carcinogens such as asbestos, diesel exhaust and some PAH, relevant for firefighters, are risk factors for lung and laryngeal cancer (24–26). Positive-pressure SCBA is the most important protection against inhalation of toxicants for firefighters, and in Norway, positive-pressure SCBA has been increasingly used since the 1980s (16).

Unfortunately, data on lifestyle habits among Norwegian firefighters were not available. Cigarette smoking is the main risk factor for lung cancer, and is also an important risk factor for laryngeal cancer alongside excess alcohol consumption (24, 27). Cancers for which smoking is a predominant risk factor are especially prone to bias by the healthy worker effect. Previous Norwegian studies reported SIR of 0.81 for lung and 0.74 for laryngeal cancer among employed men (28), and SIR of 1.15 for lung and 1.14 for laryngeal cancer among unemployed men (29) compared with the general male population. A lung cancer SIR close to unity as observed at present is in agreement with smoking habits somewhat similar to the general population, or a combined effect of less smoking and occupational exposure to carcinogens, as was suggested by the incidence pattern for laryngeal cancer.

### Mesothelioma

The elevated incidence of mesothelioma found in our study is similar to that found previously for Norwegian firefighters (SIR 2.78, 95% CI 1.02–6.06) (21), and is in line with findings from recent meta-analyses (19, 20). Inhalation of asbestos dust is the main cause of mesothelioma (30). Consistent with the frequently seen 30- to 40-year latency period for mesothelioma development following first asbestos exposure (30), we observed elevated incidence of mesothelioma among firefighters observed ≥40 years since first employment (6 cases).

Though the use and import of asbestos practically ended in Norway in the late 1970s (31), the risk of exposure remains a concern during firefighting in older burning and collapsing buildings. More cases than expected were observed across all periods of first employment, although the CI were wide and SIR based on small numbers. Stratification by employment duration suggested elevated incidence with employment duration ≥30 years, demonstrating that risk of asbestos exposure and mesothelioma may be increased with prolonged employment.

### Skin cancer

Overall, we observed more cases of cutaneous melanoma than expected, and near expected number of cases of non-melanoma skin cancer. Elevated incidence of melanoma among firefighters has previously been reported in the meta-analysis by Jalilian et al (20) and the Nordic study (21). The latter also reported elevated incidence of non-melanoma skin cancer (21). However, country-specific studies across Scandinavia have had heterogeneous findings, reporting suggested increased risk for melanoma (32), decreased risk of melanoma (33), and increased risk of non-melanoma skin cancer with no positive association to work duration (34). No clear risk patterns were observed from the present stratified analyses.

Ultraviolet radiation exposure and host pigmentation factors are major risk factors for both melanoma (35) and non-melanoma skin cancer (36). In addition, exposure to PCB can cause melanoma (37), and PAH-containing exposures such as coal tar pitch, mineral oils, and soot are classified as human carcinogens causing non-melanoma skin cancer (17). Variability in levels of PAH in skin wipe samples between firefighting job assignments (5) suggest that dermal exposures can vary widely, potentially explaining some of the observed variability in risk.

### Urinary tract and kidney cancer

Overall, there were more cases than expected of urinary tract cancer (excluding the kidney) among the fire­fighters in our cohort. Upon stratification, we observed elevated incidence among firefighters with first employment before 1950 as well as among those with ≥40 years since first employment. Bladder cancer is the predominant urinary tract malignancy, and our findings are partially in line with findings from recent meta-analyses demonstrating elevated incidence of bladder cancer among firefighters (19, 20).

Occupational exposure to mixtures containing benzo[*a*]pyrene has been associated with bladder cancer (1, 26), as has exposure to diesel exhaust (8). The risk patterns we observed may be an indication that these exposures were greater among firefighters with earlier periods of first employment. Improvements through recent decades in the quality, use, and maintenance of SCBA and personal protective equipment and the installment of local exhaust removal systems in Norwegian fire departments may have contributed to reduced exposures (16), and may correspond to the near-expected incidence observed among firefighters who began working more recently.

Nonetheless, recent studies have detected significantly elevated levels of PAH in the urine of firefighters after compared to before firefighting (11, 13), and accumulating PAH in the urine among firefighting instructors with repeated daily firefighting exercises (38) despite SCBA use during active firefighting. These exposures were determined to have occurred via dermal absorption or via inhalation when SCBA was not used, and may remain relevant modes of exposure to carcinogens for firefighters in the present study, as well. As latency-based trends were suggested alongside period-based trends and there were few (N=3) cases of urinary tract cancers in the stratum with <20 years since first employment, it may also be that these cancers have yet to present among firefighters with relatively recent first employment.

In line with the Nordic study (21) and the meta-analyses (19, 20, 23), no elevated risk for kidney cancer was found among firefighters in the present study, nor did any patterns emerge from the stratified analyses.

### Lympho-hematopoietic cancers

We did not find elevated incidence of any subgroup of lympho-hematopoietic cancers, nor did any patterns arise from the stratified analyses. In line with our findings, meta-analyses also did not demonstrate elevated incidence of these cancers among firefighters (19, 20, 23), though elevated mortality and elevated summary risk estimates based on mortality studies have been reported for multiple myeloma (23) and non-Hodgkin lymphoma (20, 23). The Nordic study showed elevated incidence of multiple myeloma only among those >70 years of age at follow-up (21).

Exposure to carcinogens such as benzene, 1,3-butadiene, formaldehyde, styrene, and diesel exhaust, all relevant to firefighters, have established or suspected associations with specific groups of lympho-hematopoietic cancers (1, 18). Firefighters in Norway may not be at elevated risk for these cancers, or this risk may have dissipated over time alongside improved protection from associated exposures. Alternatively, the present findings may be related to a low number of cases and thus a lower statistical power for detecting small excesses in these cancers.

### All sites combined

Overall, incidence of all cancers combined was significantly elevated by 15% among firefighters in the present study. Our findings are in line with the study on Nordic firefighters which also reported elevated incidence of all sites combined (21), but contrast earlier meta-analyses and other Scandinavian country-specific studies which reported at level with expected (19, 20, 23, 32, 34) or decreased (33) incidence of all sites combined.

We also observed a non-significant 10% elevated incidence for the group exposure-associated sites, which combined the sites of *a-priori* interest with established associations with specified exposures. The trends in the stratified analyses for exposure-associated sites combined were slightly more pronounced than the trends for all sites combined, though both followed similar patterns with most prominently elevated risk among those with first employment before 1950, with ≥40 years since first employment, and with employment duration ≥30 years.

Exposure to 2,3,7,8-tetrachlorodibenzo-*para*-dioxin, a group 1 carcinogen detected at the fire scene, has been associated with elevated risk of all sites combined (1, 26). In addition, firefighters’ exposures are diverse and variable, and represent chronic and simultaneous exposure to a multitude of site-specific carcinogens and other chemicals. Potential synergistic, cumulative, and/or otherwise interactive effects of exposure to a variety of compounds not classified as carcinogens, but still with the ability to change cells in the same direction (39), are difficult to assess and are not yet well established (9). However, exposure to such a “cocktail” of chemicals, even at low levels, has been hypothesized to have effects similar to those from carcinogens (9), and may have contributed to the elevated incidence of exposure-associated sites or all sites combined. Differences with previous studies reiterate that firefighters’ exposures and risks are complex and variable.

### Strengths and limitations

The population in Norway was 3.6 million in 1960 (median age 33 years), and 5.3 million in 2018 (median age 39 years) (40). Given the population size and limited number of firefighters in Norway, the study sample identified from the cohort is relatively large, including almost exclusively full time professional firefighters from the largest departments over a long follow-up period. The present study sample includes 3881 male firefighters actively employed for an average of more than 20 years between 1950 and 2018, compared to the 2579 Norwegian self-reported firefighters included in a previous study identified by census data from 1960, 1970 and 1980 (21). With limited between-department transfers and low emigration rates in our cohort, the likelihood that we lacked employment history and/or incidence data that could essentially change the observed results is low. Further, with the high degree of coverage and strict quality control measures at the CRN (22), we can expect that the incidence rates for the cohort and reference population are valid. However, risk estimates based on few observed or expected cases may still be vulnerable to random variation, and the low number of cases for some sites in the cohort limits the statistical power and the precision in the estimates.

As firefighters are required to be in good health at first employment and to stay in good health during their work, a healthy worker effect may have biased our results. While cancer outcomes are usually not considered to be strongly affected by this and the effect diminishes over time, previous studies have provided a complex picture of the potential impact of the healthy worker effect in Norway, particularly for cancer sites where cigarette smoking is a predominant risk factor (28, 29). If present, the effect may disguise true elevated risks from occupational exposure to carcinogens and contribute to underestimations of firefighters’ occupational cancer risk. Unfortunately, we had no information about lifestyle factors of firefighters.

Like almost all other studies on firefighters’ cancer risk, this study is limited by its lack of specific data on exposures as only exposure surrogates were used. The carcinogens that firefighters can be exposed to through their work are complex and dynamic and are particularly difficult to measure during active firefighting duties. Further, fire contents and exposures may vary regionally, and recent measurements may not reflect historical exposures or variation in use of protective equipment. This is partly compensated by the focus on a quite homogenous group of fulltime workers that can reasonably be expected to have had principally common occupational exposures. Studies with more detailed exposure assessments would be valuable for a better understanding of differences in risk.

### Concluding remarks

We found increased incidence of all cancer sites combined among firefighters in the Norwegian Fire Departments Cohort compared to the general population, and increased incidence of the group of exposure-associated sites combined was suggested overall. Incidence of urinary tract cancer was increased among those with first employment in earlier periods and with longer time since first employment. With longer duration of employment and with longer time since first employment, we also observed higher incidence of laryngeal cancer and mesothelioma. Our findings are partly in line with those from previous meta-analyses showing elevated incidence of bladder cancer and mesothelioma and of all sites combined in the study on Nordic firefighters. This suggests that the elevated risks observed are associated with firefighters’ occupational exposures. Differences in risk by period of first employment may primarily reflect changes in exposures from improved quality and use of personal protective equipment.

## Supplementary material

Supplementary material
