# Supplementary material for: Cancer incidence in sites potentially related to occupational exposures: 58 years of follow-up of firefighters in the Norwegian Fire Departments Cohort
Source: Scand J Work Environ Health. 2022 Mar 31;48(3):210–9. doi: 10.5271/sjweh.4009 (PMC9523464; doi:10.5271/sjweh.4009)
Supplement: Supplementary material [file SJWEH-48-210-S001.pdf]

Cancer incidence in sites potentially related to occupational exposures: 58 years of follow-up of firefighters in the Norwegian Fire Departments Cohort[1]

by Niki Marjerrison, MPhil,[2] Jarle Jakobsen, MD, Tom K Grimsrud, PhD, Johnni Hansen, PhD, Jan Ivar Martinsen, Karl-Christian Nordby, PhD, Marit B Veierød, PhD, Kristina Kjærheim, PhD

1. Supplementary material
2. Correspondence to: Niki Marjerrison, Department of Research, Cancer Registry of Norway, P.O. Box 5313, 0304 Oslo, Norway. [E-mail: niki.marjerrison@kreftregisteret.no]

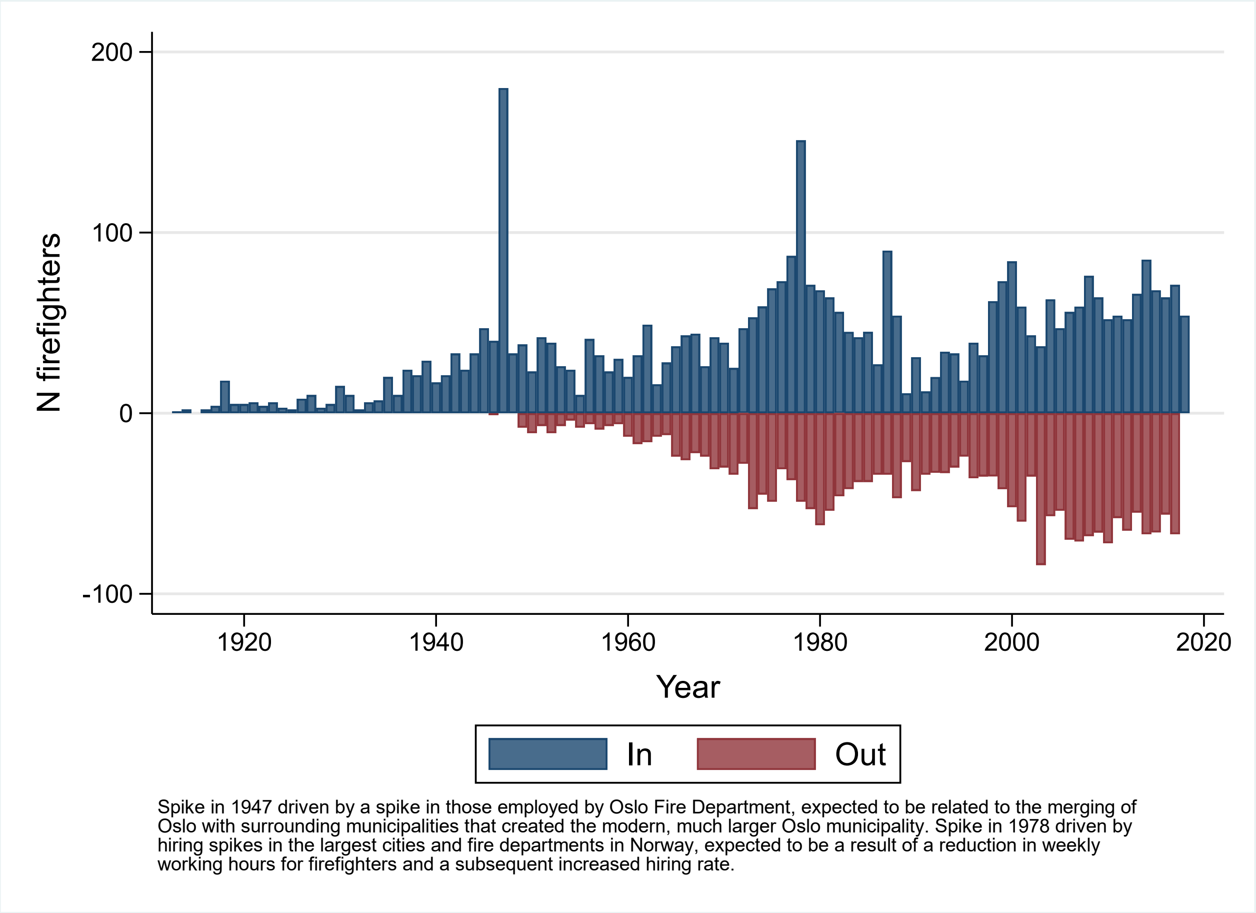

Supplementary figure S1. Number of employees starting and stopping employment per year in the Norwegian Fire Departments Cohort, 1913–2018.

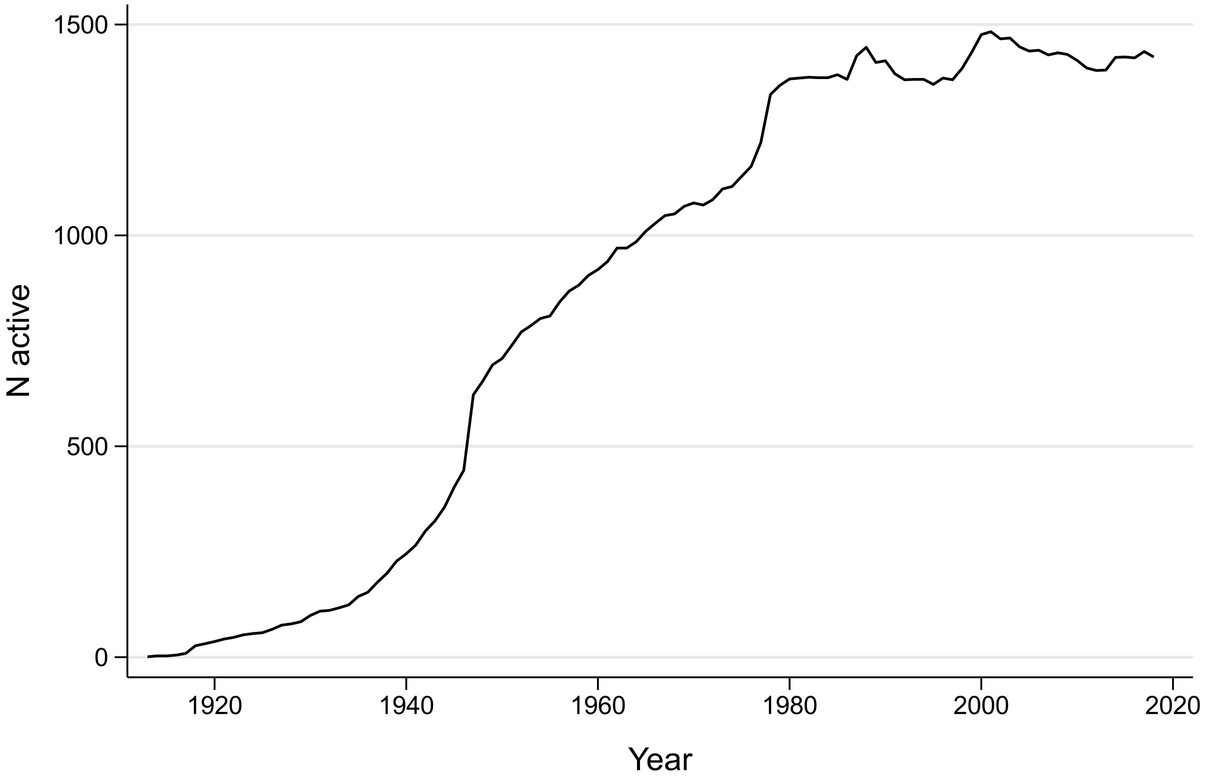

Figure depicts the number of active employees at the 15 fire departments in the Norwegian Fire Departments Cohort who were employed during the period 1950–2018 and alive during the period 1960–2018, with the earliest start of employment in 1913.

Supplementary figure S2. Number of active employees per year in the Norwegian Fire Departments Cohort, 1913–2018.
